# Effects of exercise snacks combining resistance training and Wuqinxi on older adults with possible sarcopenia in nursing homes: protocol for a randomized controlled trial

**DOI:** 10.3389/fpubh.2026.1960282

**Published:** 2026-10-14

**Authors:** Jialing Wang, Tianliang Ji, Xiaorong Dong, Xindi Shao, Yuan Zhang, Yihan Wang, Xueyan Zhang

**Affiliations:** 1 School of Nursing, Jilin University, Changchun, China; 2 Department of Cardiovascular Medicine, The First Hospital of Jilin University, Changchun, China

**Keywords:** exercise snacks, nursing homes, possible sarcopenia, randomised controlled trial, resistance training, Wuqinxi

## Abstract

**Aim(s):**

To evaluate the effectiveness and feasibility of a Fogg Behaviour Model-based exercise-snack intervention combining resistance training and Wuqinxi for improving physical function among older adults with possible sarcopenia living in nursing homes.

**Design:**

This study is a single-centre, assessor-blinded, two-arm randomised controlled trial protocol.

**Methods:**

Older adults with possible sarcopenia will be recruited from a nursing home in Changchun, China, and randomly allocated in a 1:1 ratio to either a 12-week exercise-snack intervention group or a wait-list control group. Guided by the Fogg Behaviour Model, the intervention combines resistance training and Wuqinxi delivered through short, repeated bouts of exercise to enhance exercise motivation, ability and adherence. Outcome assessments will be conducted at baseline and at weeks 4, 8 and 12. The primary outcome is physical function measured using the Short Physical Performance Battery. Secondary outcomes include upper- and lower-limb muscle strength, dynamic and static balance ability, cognitive function, exercise adherence and adverse events. Data will be analysed using linear mixed-effects models, with group, time and the group × time interaction included as fixed effects and participant-specific random intercepts.

**Results:**

Participant recruitment and data collection are ongoing. Study findings will be disseminated following completion of the trial and final analyses.

**Conclusion:**

This trial will provide evidence regarding the feasibility, safety and effectiveness of a Fogg Behaviour Model-based exercise-snack intervention for improving physical function among older adults with possible sarcopenia in nursing homes.

**Trial registration:**

Chinese Clinical Trial Registry (ChiCTR2600126344).

## Background

With the extension of life expectancy, population ageing has become an increasingly pressing global concern. In China, adults aged 60 years and older accounted for 18% of the total population in 2020, and this proportion is projected to rise to 39% by 2050 (1). Epidemiological evidence has demonstrated that adults over 50 years of age experience progressive muscle loss of 1–2% annually (2). Sarcopenia is defined as an age-related syndrome characterised by the progressive decline in skeletal muscle mass, muscle strength and physical function. It is estimated that sarcopenia affects 10 to 16% of older adults worldwide (3). Notably, this prevalence is substantially higher among nursing home residents, with reported rates ranging from 31.4 to 38.3% (4). Mounting research has established that sarcopenia is closely associated with a range of adverse health outcomes, including accelerated progression of cardiovascular disease, elevated risks of falls and all-cause mortality, and compromised quality of life and healthspan among older adults (5). However, findings from a large-scale longitudinal study have shown that sarcopenia is a dynamic condition: individuals can transition between non-sarcopenic, possible sarcopenic and sarcopenic states through natural progression or targeted interventions, which may reverse sarcopenia to possible sarcopenic or even non-sarcopenic status (6). Therefore, early identification of individuals at risk of sarcopenia and the timely implementation of appropriate interventions are of great importance for delaying disease progression, preventing the onset of sarcopenia, and potentially promoting its reversal. Based on this concept, the Asian Working Group for Sarcopenia 2019 (AWGS 2019) proposed the concept of possible sarcopenia (7), defined as an early risk state characterised mainly by reduced muscle strength and/or impaired physical performance, which does not meet the diagnostic criteria for confirmed sarcopenia. Furthermore, the AWGS 2025 guideline (8) was updated: the physical performance indicator was removed, and low handgrip strength was retained as the core diagnostic criterion on the premise that muscle mass is not measured. Physical performance indicators are no longer used for diagnosis and are only adopted as outcome indicators after intervention. This concept emphasises advancing the intervention window to enable early identification and intervention before progression to confirmed sarcopenia. Therefore, compared with therapeutic interventions for older adults with established sarcopenia, preventive interventions targeting older adults with possible sarcopenia can block the onset and progression of sarcopenia at an earlier stage. Accordingly, this study intends to recruit older adults with possible sarcopenia and implement a comprehensive exercise intervention aiming to improve their physical performance and muscle strength. This study can not only provide new evidence for the early prevention and management of sarcopenia, but also help improve the health status of older adults, reduce the consumption of medical resources, and alleviate the social and medical burdens caused by sarcopenia.

The 2025 Asian Working Group for Sarcopenia Consensus (8) explicitly identifies multimodal exercise combined with nutritional optimisation as the core intervention strategy for muscle health in individuals with sarcopenia. At present, exercise prescriptions for sarcopenia mainly focus on resistance exercise, which is often combined with aerobic exercise, balance training and other modalities. Resistance training can effectively enhance muscle strength (9). Combined training consists of multiple training modalities and can produce differential improvements in muscle strength, balance, gait speed and overall physical performance. Its specific advantages depend on the composition, intensity and duration of the training programme (10), providing theoretical support for clinical intervention and daily rehabilitation. Nevertheless, single-mode resistance training has notable limitations. It does not adequately improve flexibility and balance, is associated with poor long-term exercise adherence and has limited efficacy in fall prevention. As a traditional Chinese health-preserving exercise, Wuqinxi involves gentle movements and emphasises harmony between body and mind, making it highly compatible with the physiological characteristics of older adults. It is engaging and easy to maintain, and can effectively improve balance ability and cognitive function in older adults (11). A 24-week randomised controlled trial also demonstrated that Wuqinxi can improve dynamic and static balance in women with a history of falls (12). Another 12-week Wuqinxi intervention found that the intervention group showed significantly better cognitive function than the stretching group (13). Although resistance training combined with Wuqinxi has theoretical advantages, older adults with sarcopenia often have reduced muscle mass, muscle weakness and physical dysfunction. The long duration and high intensity of conventional combined exercise may easily lead to fatigue and poor exercise adherence.

Therefore, appropriately reducing exercise intensity and shortening the duration of each exercise session may represent a more suitable training model. Accordingly, this study intends to integrate the core strengths of resistance training and Wuqinxi, and to adopt exercise snacks as the delivery mode to develop a fragmented, low-intensity combined intervention. This approach retains the muscle-strengthening effects of resistance training and incorporates the benefits of Wuqinxi for balance and cognitive function, while accommodating the physical limitations of older adults with sarcopenia. Exercise snacks are defined as repeated bouts of exercise lasting less than 1 min each, scattered throughout the day (14). A complete training session may consist of multiple bouts of such short-duration exercise, with each bout lasting less than one minute; thus, the total duration of the entire training session could be longer than one minute. As an emerging physical activity mode, exercise snacks are characterised by fragmented, short-duration and environment-flexible activity, which aligns with the practical constraints of older adults who are unable to engage in prolonged continuous exercise because of age-related physiological changes, physical limitations and environmental factors. Notably, exercise snacks have been shown to be a highly feasible, safe and acceptable form of exercise for older adults (8). Moreover, this exercise mode has been shown to improve lower extremity muscle function and muscle mass (15), as well as balance, mobility (16) and cognitive function (27) in healthy older adults. Therefore, exercise snacks hold promise as an innovative exercise intervention for older adults with sarcopenia.

Although exercise snacks confer substantial physical benefits, developing a consistent exercise habit remains challenging for older adults with sarcopenia. The Fogg Behaviour Model (FBM), a classic behaviour-change framework proposed by BJ Fogg (2009), emphasises three core elements for behaviour enactment: motivation, ability and prompt. Motivation refers to the psychological drive that impels individuals to adopt a specific behaviour. Ability denotes the practical feasibility of an individual performing the behaviour; behaviour requires not only the willingness to act but also the capacity to do so. A prompt is an external cue that triggers behaviour initiation; when motivation and ability are sufficient, prompts can evoke action. To date, this behavioural model has been widely applied in studies designed to facilitate behaviour change. A recent systematic review highlighted the FBM as an effective framework for promoting health behaviour modification across diverse domains. Through the systematic integration of motivation, ability and prompts, FBM-based interventions have demonstrated promising outcomes (17). Therefore, the intervention protocol in this study will be developed based on the Fogg Behaviour Model, targeting the three core elements of motivation, ability and prompt to promote exercise behaviour among older adults with sarcopenia, thereby enhancing exercise adherence and intervention efficacy.

## Methods

### Study design and ethical statement

This randomised controlled trial (RCT) aims to evaluate an exercise-snack intervention combining resistance training and Wuqinxi among older adults with sarcopenia in nursing homes. The study flowchart is shown in Figure 1, and the study procedure is shown in Table 1. Ethical approval was obtained from Committee of the School of Nursing, Jilin University (Approval No. 2025112712) and will be conducted in strict accordance with the Declaration of Helsinki. The trial was registered with the Chinese Clinical Trial Registry on 8 June 2026 (registration number: ChiCTR2600126344). The first participant was enrolled on 15 June 2026, and the trial is currently in the intervention phase.

**Figure 1:**
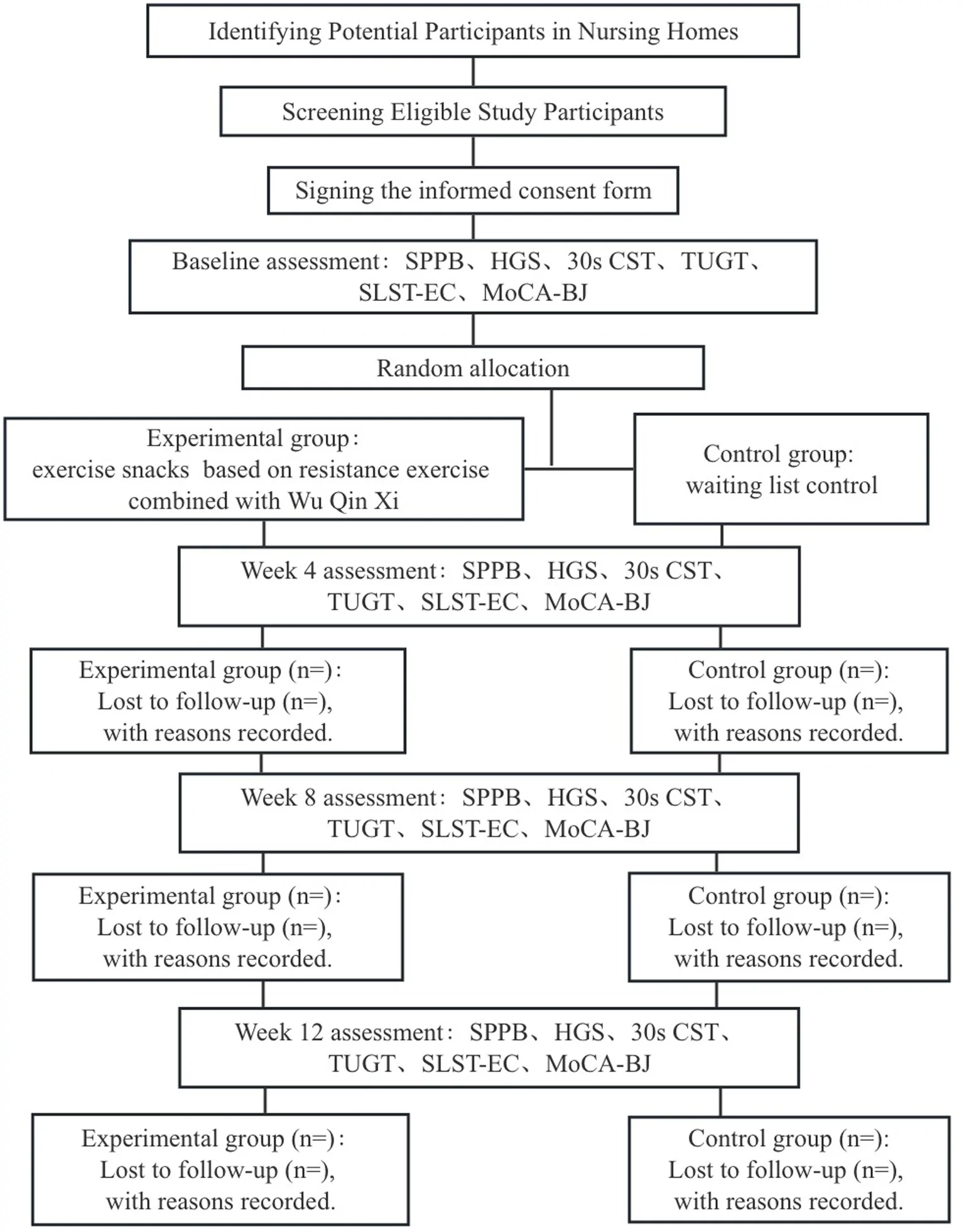
Flowchart of participant enrolment and data collection. SPPB, Short Physical Performance Battery; HGS, handgrip strength. 30-s CST: 30-s chair stand test; TUGT, Timed Up and Go Test; SLST-EC, Single-Leg Stand Test with Eyes Closed; MoCA-BJ, Beijing version of the Montreal Cognitive Assessment.

**Table 1:** The study procedure.

Stage/time point	T-1	T0	T1	T2	T3
Enrolment session					
Eligibility review	√				
Informed consent	√				
Intervention session					
Intervention group			√	√	√
Control group					
Assessment session					
General information		√			
SPPB		√	√	√	√
HGS		√	√	√	√
30-s CST		√	√	√	√
TUGT		√	√	√	√
SLST-EC		√	√	√	√
MoCA-BJ		√	√	√	√
Exercise adherence			√	√	√
Adverse events			√	√	√

T-1 = recruitment and screening; T0 = baseline, before intervention initiation; T1 = week 4 after intervention initiation; T2 = week 8 after intervention initiation; T3 = week 12 after intervention initiation.

SPPB, Short Physical Performance Battery; HGS, handgrip strength; 30-s CST, 30-s chair stand test; TUGT, Timed Up and Go Test; SLST-EC, Single-Leg Stand Test with Eyes Closed; MoCA-BJ, Beijing version of the Montreal Cognitive Assessment; SLST-EC, Single-Leg Stand Test with Eyes Closed.

### Participant selection

Convenience sampling will be used to recruit older adults residing in a nursing home in China. The inclusion criteria are as follows: (1) aged ≥60 years; (2) meeting the diagnostic criteria for possible sarcopenia specified in the Expert Consensus on Diagnosis and Treatment of Sarcopenia in Older Adults in China (2025 Edition): for individuals aged ≥65 years, handgrip strength <28 kg in males and <18 kg in females; for individuals aged 60–64 years, handgrip strength <34 kg in males and <20 kg in females; (3) able to complete the Short Physical Performance Battery (SPPB), with no component score of 0; and (4) providing informed consent and voluntarily agreeing to participate. Participants will be excluded if they: (1) have absolute contraindications to exercise, such as deep vein thrombosis and/or coagulation disorders; (2) take medications that affect cognitive function; (3) have participated in other research projects targeting improvement in physical capacity or cognitive function within the previous 6 months; or (4) have a regular exercise habit, defined as engaging in vigorous-intensity exercise at least twice per week or moderate-intensity exercise at least three times per week, with each session lasting >30 min.

### Sample size

The sample size was estimated *a priori* using G*Power 3.1 based on a two-group mixed-design repeated-measures analysis of variance (ANOVA), with the calculation aimed at detecting the group × time interaction effect for the primary outcome, the Short Physical Performance Battery (SPPB). Based on previously published studies of a similar nature (18, 26) and the results of our pilot study, a conservative moderate effect size of partial eta squared (ηp^2^) = 0.04 (Cohen’s *f* = 0.20) was assumed. The significance level was set at *α* = 0.05, with a statistical power (1 − *β*) of 0.80, four repeated-measures time points, a correlation of 0.50 between repeated measures, and a nonsphericity correction factor (ε) of 1.0. Assuming an anticipated dropout rate of 20%, the required total sample size was estimated to be 45 participants. Although the primary analysis of this study will be conducted using a linear mixed-effects model rather than repeated-measures ANOVA, linear mixed-effects models can make use of partial observations from participants with missing follow-up visits. Therefore, the prespecified sample size is considered to remain conservative.

### Randomisation and allocation concealment

Eligible participants who meet the inclusion and exclusion criteria will be randomly allocated in a 1:1 ratio to either the intervention group or the control group using a computer-generated randomisation sequence. The randomisation process will be conducted by a researcher who is not involved in participant screening, intervention delivery, data extraction, or data analysis. As all participants will be recruited from the same nursing home, their frequent interactions with one another may result in intervention contamination. Therefore, participants’ living arrangements will be considered during the recruitment process. If multiple residents sharing the same room meet the eligibility criteria, only one resident will be enrolled, after which individual randomisation will be performed. This measure is intended to minimise potential cross-group contamination arising from shared accommodation, including mutual learning and behavioural influences between participants assigned to different study groups, while maintaining an individual-level randomisation design. To monitor potential contamination, participants in the control group will be asked at each follow-up assessment whether they have engaged in resistance exercise, Wuqinxi, other Qigong exercises, or other structured physical activities outside the study. Any such activities will be documented, including their type, frequency, and duration, where possible. Allocation concealment will be managed by an independent researcher. The randomisation sequence will be stored in a password-protected file and will remain inaccessible until participant eligibility has been confirmed and the baseline assessment has been completed. Group allocation will then be disclosed to ensure adequate allocation concealment.

### Intervention

Based on the Fogg Behaviour Model (FBM), previous intervention literature on older adults with sarcopenia and evidence on exercise snacks, a preliminary training protocol was developed and revised following expert consultation with specialists in geriatrics and rehabilitation medicine and consideration of pilot study results from our research team. The final intervention protocol was confirmed after group discussions.

The FBM emphasises three core elements for behaviour change: motivation, ability and prompt. These elements guided the design of the intervention, as described below. The specific intervention schemes are shown in Figure 2.

**Figure 2:**
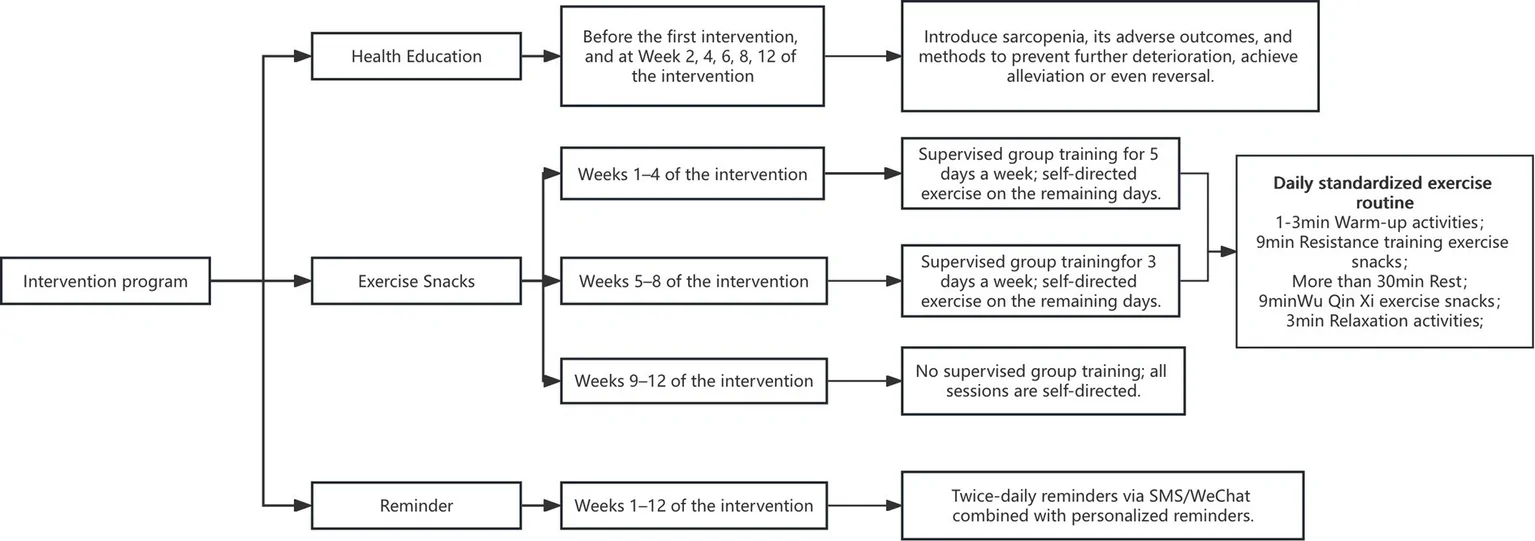
Intervention procedure.

### Motivation

Motivation refers to the psychological drive that prompts an individual to engage in a specific behaviour. In this study, it denotes the psychological impetus for older adults with sarcopenia to participate in exercise. Therefore, this study plans to conduct a baseline health education session before the first formal exercise-snack intervention, followed by additional health education sessions for participants in the intervention group every 2 weeks. Health education will be delivered through lectures in which the intervention group will view popular science videos on sarcopenia in small groups, covering the definition of sarcopenia, its clinical manifestations, associated health hazards and strategies for improving or delaying its progression.

### Ability

Ability represents the practical feasibility for individuals to carry out a specific behaviour, which can be enhanced by simplifying behavioural procedures and reducing barriers to performance. Accordingly, this study intends to improve the exercise capacity of older adults with sarcopenia in nursing-home settings via the exercise-snack paradigm. The intervention will be implemented for 7 days per week, consisting of two daily exercise-snack training units: one session of exercise-snack resistance training and one session of exercise-snack Wuqinxi training. Each training unit lasts 9 min. Referring to guidelines on accumulated short-bout exercise (19), the interval between the two training sessions should be no less than 30 min. Exercise-snack resistance training comprises five movements: standing exercise, seated resistance-band press, seated alternating knee extension, calf raise, and sit-to-stand training. Exercise-snack Wuqinxi consists of five forms imitating the tiger, deer, ape, bear, and bird. Each movement has two difficulty levels, with training initiated at Level 1. Participants will follow individualised, staged, and progressive exercise principles and select appropriate exercise intensity or velocity according to their own tolerance. In principle, exercise intensity is targeted at a score close to 5 on the Borg CR-10 Rate of Perceived Exertion scale. After participants adapt to the current training intensity and meet pre-set progression criteria, they may advance to the next level upon instructor assessment. Each movement lasts approximately 1 min, followed by a 1-min rest between consecutive movements. The total duration of each exercise-snack resistance-training or Wuqinxi-training unit is around 9 min. Detailed movements and training requirements are presented in Table 2.

**Table 2:** Detailed guidelines for the exercise-snack intervention combining resistance training and Wuqinxi.

Exercise	Level	Specific procedure	Progression criteria
Standing (core, thighs and balance)	Level 1	Heel-to-toe standing: Stand with the toes of one foot touching the heel of the other foot in a straight line for 1 min.	Starting level
	Level 2	Standing (feet shoulder-width apart), hands on hips (can use support for balance); Lift one leg, maintain balance, and lower it back down. Try to hold for 1 min.	Level 1 movements can be completed smoothly without handrail assistance; loss-of-balance episodes shall not exceed 5 times within 1 min.
Seated Resistance-Band Press	Level 1	Sit upright with straight lower back and upper back supported against the chair. Keep chest lifted, shoulders depressed and core engaged for trunk stabilization. Raise both hands vertically overhead, with a 10-lb resistance band looped around the forearms. Pull arms downward by activating back muscles, lower slowly and controllably until upper arms are parallel to the floor. Exhale during downward pulling; inhale while returning to the starting position.	Starting level
	Level 2	Same as Level 1, but increase resistance-band poundage.	Level 1 movements are smooth and controllable without external assistance, with more than 20 repetitions completed within 1 min.
Seated alternating knee extension (thighs and core)	Level 1	Place hands naturally on thighs (or lightly hold chair sides for balance only), knees bent 90° (thighs and calves at right angle); engage core slightly, gaze forward. Tighten core, slowly lift left leg, gradually extend knee (point toes upward), extend until “knee nearly straight but not locked” (keep 5–10° slight flexion to avoid hyperextension). Hold at the top position for 5–10 s.	Starting level
	Level 2	Add ankle weights, increasing by 0.5 kg each time after adaptation. Place hands naturally on thighs (or lightly hold chair sides for balance only), knees bent 90° (thighs and calves at right angle), engage core slightly, gaze forward. Tighten core, slowly lift left leg, gradually extend knee (point toes upward), extend until “knee nearly straight but not locked” (keep 5–10° slight flexion to avoid hyperextension). Hold at the top position for 5–10 s.	Level 1 action is controlled well and stable, no chair support needed, and can hold the top position for 10 s to progress to Level 2.
Heel raises (ankle stability)	Level 1	Standing heel raises: Stand (feet shoulder-width apart), hands on hips (can use support for balance). Rise onto toes (heels off ground) and lower back down. Repeat as many times as possible within 1 min.	Starting level
	Level 2	Single-leg heel raises: Stand (weight on one leg, other leg slightly lifted). Rise onto toes of support leg (heel off ground) and lower back down, 30 s per side, hands on hips (can use support for balance). Alternate sides within 1 min.	Level 1 action is smooth and well-controlled with no swaying. ≥45 repetitions completed in 1 min.
Sit-to-stand (thighs)	Level 1	Free-arm sit-to-stand: Sit on a dining chair (upright posture). Place hands naturally, use arm strength to stand up and sit down, repeating the movement. Maintain a steady pace, repeat as many times as possible within 1 min. Chair support is allowed.	Starting level
	Level 2	Crossed-arm sit-to-stand: Sit on a dining chair (upright posture). Cross arms over chest (no arm strength allowed), stand up and sit down using only leg strength, repeating the movement. Repeat as many times as possible within 1 min.	Level 1 movements are smooth and controllable without external assistance, with more than 25 repetitions completed within 1 min.
Tiger Play	Level 1 Tiger Raise	Form “tiger claws” (fingers spread and flexed, palms convex) in front of the abdomen. Raise both hands slowly upward along the midline of the body; turn palms upward at chest level as if lifting a heavy object, until reaching above the head. Eyes follow the hands. Change back to “tiger claws” and pull downward along the same path to the front of the abdomen.	Starting level
	Level 2 Tiger Pounce	Lift and extend both hands sideways and forward; bend the upper body forward and press downward with “tiger claws.” Bend knees and squat, draw in the abdomen and tuck the chest; retract both hands to the waist. Extend knees and push hips forward, arch the abdomen and tilt backward; simultaneously swing both hands forward and upward in an arc, simulating a tiger pouncing on prey. May step forward with the lower limbs to perform the pouncing movement.	The full set of movements can be completed independently for 4 repetitions.
Deer Play	Level 1 Deer Push	Slightly bend both knees; shift body weight to the right leg, step the left foot forward-leftward with heel touching the ground. Turn the body leftward; bend the right elbow forward and draw a horizontal arc leftward with fingertips forward; bend the left elbow backward with the dorsum of the hand against the lower back. Eyes follow the right hand; when turning left, gaze to the left-rear, imitating a deer looking back. Return and switch sides.	Starting level
	Level 2 Deer Run	Step the left foot forward; raise both arms forward to shoulder level. Shift weight backward, sit back with the right leg bent and the left leg extended, toes raised; rotate both arms inward, tuck the chest and draw in the abdomen to form “horizontal arch” and “vertical arch” in the back. Shift weight forward, straighten the right leg and bend the left leg; rotate both arms outward and extend forward. Switch sides.	Level 1 movements can be completed independently for 4 repetitions.
Ape Play	Level 1 Ape Lift	Place hands in front of the body, fingers straight and spread, then quickly clench into “ape hooks” (wrist flexed, fingers gathered). Simultaneously rise onto toes, contract the anus, tuck elbows, shrug shoulders, compress the chest and draw in the abdomen; turn head leftward with alert gaze. Relax, turn hands to palms and press downward to the front of the abdomen, fully relaxing the body. Turn head to the other side and repeat.	Starting level
	Level 2 Ape Pluck	Step backward leftward, draw the left hand backward in an arc, bend elbow and support at the left waist; swing the right hand forward and upward as if climbing a branch. Step the right foot forward-rightward, follow with the left foot into a T-stance. Extend the left hand quickly forward under the right arm, form “ape hook” to imitate fruit picking; retract the right hand quickly to below the left elbow. Eyes follow the left hand, focusing when picking fruit. Switch sides.	Level 1 movements can be completed independently for 4 repetitions.
Bear Play	Level 1 Bear Rotate	Form loose fists into “bear palms” and place below the abdomen. Using the waist and abdomen as the axis, rotate the upper body clockwise in a vertical circular motion. The fists follow the path: right ribcage → upper abdomen → left ribcage → lower abdomen. Repeat counterclockwise.	Starting level
	Level 2 Bear Sway	Lift the left hip to raise the left leg, then step forward-leftward. Shift body weight forward, straighten the right leg and bend the left leg; simultaneously push the left arm forward and swing the right arm backward. Sit back with weight shifted, bend the right leg and straighten the left leg; turn the body rightward and push the left arm inward and forward. Switch sides.	Level 1 movements can be completed independently for 10 repetitions to the left and 10 repetitions to the right.
Bird Play	Level 1 Bird Stretch	Slightly bend knees; overlap palms in front of the abdomen. Straighten legs, raise palms forward above the head with palms downward, lean slightly forward to imitate a bird stretching wings. Press palms down to the abdomen, then swing them backward; bend the upper body forward and lift one leg backward to form a reverse arch. Rise and return.	Starting level
	Level 2 Bird Fly	Extend both arms sideways to shoulder level, lift one knee upward. Lower both arms and the lifted leg to return to standing or feet together. Lift the other knee and raise both arms upward, imitating a bird flapping wings to soar. Move up and down gently and smoothly.	Level 1 movements can be completed independently for 4 repetitions.

### Instructor training and standardised implementation

All research staff involved in intervention delivery will receive unified standardised training before study commencement. Training content includes the study protocol, intervention implementation procedures, standard operation of resistance-training and Wuqinxi movements, exercise-intensity regulation, movement-quality requirements, principles for individualised adjustment, completion and verification of exercise logs, as well as identification and emergency management of adverse events. Training will be delivered by research personnel with backgrounds in exercise training or rehabilitation, combining theoretical lectures, movement demonstrations, and hands-on practice. All instructors must complete the training and pass operational assessments before participating in intervention delivery. A unified intervention manual and demonstration materials will be used throughout the study to ensure consistent implementation across instructors.

### Standardised training procedures

Each training session will follow a unified workflow consisting of four phases: pre-training warm-up, formal training, cool-down, and post-training documentation. A 1–3 min warm-up will be performed at the beginning of each session, followed by approximately 9 min of formal training according to the protocol. Upon completion of one training unit, participants will rest for at least 30 min before performing the second exercise-snack session. Appropriate cool-down activities will be conducted after the second session. Participants will then complete training records including actual training content, difficulty level, and other required information. The daily standardised exercise workflow is illustrated in Figure 2.

### Staged guidance and autonomous training

Staged guided and autonomous training will be adopted to help participants gradually adapt to movements and build independent-exercise capacity. During Weeks 1–4, standardised intervention staff will deliver group-guided training 5 days per week; participants will complete training independently in the nursing-home activity room on the remaining 2 days. During Weeks 5–8, group-guided training will be provided 3 days per week, and participants will perform independent training on the other 4 days. Weeks 9–12 constitute the full autonomous-training phase with no on-site movement guidance provided. All autonomous training will take place in the nursing-home activity room with trained safety observers on-site. Safety observers will not provide movement guidance; they will only monitor participants’ exercise status, identify potential adverse events in a timely manner, and guide participants to fill in exercise logs upon training completion. In exercise logs, participants will record training dates, the actual number of completed exercise-snack bouts, and training levels. Researchers will collect and check logs weekly and calculate the actual training dose completed by participants. Non-compliance with scheduled training will be documented together with potential reasons.

### Intervention adjustment and progression rules

To balance participants’ individual physical conditions and protocol consistency, individualised adjustments to training difficulty are permitted within pre-specified ranges. Participants will start training at Level 1 in principle. They may progress to Level 2 upon instructor assessment if they can complete current movements safely and correctly while meeting pre-set intensity requirements. Adjustments to training difficulty are limited to pre-defined movement levels, support modalities, and load ranges specified in the protocol. For participants with poor balance or difficulty completing movements, chair backs, handrails or other stable supports may be used during training, and a lower difficulty level may be maintained according to practical conditions. All adjustments to training levels, supportive measures, and other protocol-allowed modifications shall be documented in intervention record forms.

### Prompt

A prompt is an external cue that triggers individuals to initiate specific behaviours. Therefore, this study will combine daily exercise reminders and personalised prompts to facilitate participants’ adherence to daily training. Staff on each floor of the nursing home will notify participants to attend training in the activity room every day. Furthermore, personalised prompts will be set according to each participant’s daily routines and behavioural habits. Exercise training will be integrated with fixed daily activities to embed physical activity into participants’ original lifestyles and improve intervention adherence.

### Safety management

Given that this study involves exercise intervention, potential safety risks exist during training. Accordingly, one exercise-trained researcher and two to three nursing postgraduates will provide on-site guidance and safety surveillance during all group-guided sessions, with special attention paid to participants with poor balance or unstable centre-of-gravity control. During autonomous-training phases, participants will perform training independently in clean, spacious, safe activity rooms selected by researchers under the supervision of trained safety observers. Observers will not give movement guidance; they will monitor participants’ exercise status to detect adverse events promptly and guide participants to complete exercise logs after training. Handrails or stable objects may be used for support when performing movements associated with balance risks. Participants shall stop exercising immediately, take rest, and report to the research team promptly if any physical discomfort occurs during training.

### Control group

During the intervention period, the control group will remain on a wait-list and will receive no additional exercise-snack resistance training, exercise-snack Wuqinxi or other intervention measures. Instead, they will maintain their original lifestyle and daily routines. Measures to avoid cross-contamination will be implemented: the control group will be informed not to participate in any other resistance training or traditional health-preserving qigong training during the study period. After completion of the intervention and data collection, participants in the control group will be offered the same intervention as the intervention group, according to their own willingness.

### Blinding

Because of the substantial differences between the intervention measures, blinding of participants and intervention implementers will not be feasible. However, data collectors and statistical analysts will be blinded to group allocation to reduce measurement and analytical bias.

### Outcome measurements

All participants will undergo four assessments: baseline (T0), before randomisation; Week 4 (T1); Week 8 (T2); and Week 12 (T3) after initiation of the intervention.

### Primary outcome

The primary outcome selected in the present study is physical function, which is recommended as a key outcome indicator for individuals with sarcopenia in the 2025 Asian Working Group for Sarcopenia Consensus.

#### Physical function

Physical function will be assessed using the Short Physical Performance Battery (SPPB), recommended by the Asian Working Group for Sarcopenia (AWGS). The SPPB consists of three components: the 4-metre walk test, static balance test and chair stand test, which reflect participants’ walking speed, static balance ability and lower limb muscle strength, respectively. Each component is scored on a 4-point scale, with a total score ranging from 0 to 12; higher scores indicate better physical function. The SPPB is a useful tool for assessing physical function in older adults with sarcopenia (20).

### Secondary outcomes

#### Physical outcomes

##### Upper limb muscle strength

Grip strength of the dominant hand will be measured using a digital handheld dynamometer, with the maximum value from two trials, separated by a 5–10 s rest, recorded as the final result. Before testing, participants will be asked about hand injuries, which will be documented, and instructed to remove metal accessories, shoes, socks, mobile phones and heavy clothing. During testing, participants will stand upright with feet shoulder-width apart and shoulders slightly abducted, and will grip the dynamometer with the second joint of the index finger against the inner side, with the display facing outward, ensuring that the dynamometer does not touch the body or clothing while they squeeze with maximum effort. Test administrators may demonstrate the procedure if needed. Grip strength has been widely recognised as a reliable indicator for assessing upper limb muscle strength (21).

##### Lower limb muscle strength

Lower limb muscle strength will be assessed by counting the number of consecutive chair stands completed within 30 s. For this test, a back-supported chair with a height of 45 cm and a stopwatch will be used. Participants will sit upright on the chair with their hands crossed over their chest. Upon hearing the tester’s “start” command, they will stand up from the chair, fully straighten their body and then sit back down. The tester will ensure that participants maintain a 90-degree angle at both the hips and knees, keep their torso upright and sit fully on the chair during each sit-down phase; failure to meet these criteria will result in the repetition not being counted. The tester will initiate timing with the “start” command and record the total number of completed chair stands within 30 s. The 30-s chair stand test is a reliable and valid measure of lower body strength in older adults (22).

##### Dynamic balance ability

Dynamic balance ability will be measured using the Timed Up and Go Test (TUGT), an efficient quantitative assessment tool for functional ambulation that comprehensively reflects participants’ balance ability and physical performance during functional activities by integrating movements such as standing up, walking, turning and sitting down. Each participant will complete the test twice, and the average of the two measurements will be recorded. The specific test procedures are as follows: participants who regularly use walking aids, such as canes, in daily life will complete the test according to their usual habits, which will be clearly documented; participants will sit on the front two-thirds of a chair with a height of approximately 45 cm, maintaining a relaxed and upright upper body, back against the chair, hands resting on the thighs, feet flat on the ground and toes pointing forward; timing will start when the participant’s back leaves the chair and stop when they walk around a marker placed 3 metres away and sit back down on the chair. Test administrators may demonstrate the procedure to participants if necessary. To date, the TUG has been validated as a reliable and valid method for assessing balance and physical mobility in older adults (23).

##### Static balance ability

Static balance ability will be evaluated using the Single-Leg Stand Test with Eyes Closed (SLST-EC). Participants will maintain a standing posture with their hands hanging naturally and eyes closed. Upon the tester’s “start” instruction, participants will stand on their dominant leg without support or holding onto any equipment, slowly lifting their non-dominant leg off the ground without touching the supporting leg to maintain balance. Timing will begin when the non-dominant foot leaves the ground and stop when the supporting leg moves, the lifted foot touches the ground, or the body tilts severely or sways excessively. The test will be performed twice with a 1–2 min rest interval between trials, and the average value will be recorded. Currently, this test is widely used to assess static balance ability (24).

##### Cognitive function

Cognitive function will be assessed using the Beijing version of the Montreal Cognitive Assessment (MoCA-BJ), a validated Chinese version of the MoCA. The MoCA-BJ is a 30-point cognitive screening instrument that assesses seven cognitive domains: visuospatial/executive function, naming, attention, language, abstraction, delayed recall, and orientation. Scores are calculated according to the standard MoCA-BJ scoring criteria, with a total score ranging from 0 to 30, and higher scores indicating better cognitive function. One additional point is added for participants with 12 years of education or fewer, provided that the total score does not exceed 30. The Cronbach’s alpha coefficient of this scale is 0.85. Widely used in clinical practice in China, the MoCA-BJ is recognised as a highly sensitive tool for assessing cognitive function (25).

#### Feasibility and safety outcomes

##### Feasibility evaluation

This study will conduct a feasibility evaluation to assess the implementation of the research protocol and to provide a basis for subsequent confirmatory randomized controlled trials. The feasibility indicators and their corresponding criteria will be predefined prior to the commencement of the study, including recruitment rate, retention rate, intervention adherence, intervention fidelity, and acceptability. Recruitment feasibility will be defined as achieving ≥70% of the planned sample size within the prespecified recruitment period. Retention feasibility will be defined as ≥80% of enrolled participants completing the post-intervention outcome assessments. Intervention adherence will be evaluated based on the proportion of prescribed exercise sessions actually completed by participants relative to the total number of planned sessions, with ≥75% considered as meeting the adherence criterion. Safety feasibility: No intervention-related serious adverse events (SAEs) occur, and adverse events (AEs) do not substantially interfere with the continued implementation of the intervention. Acceptability will be measured using a 5-point Likert scale, with a mean score of ≥4 considered as meeting the acceptability criterion. Each of the above indicators will be evaluated separately, and the feasibility of the research protocol will be determined based on the prespecified threshold criteria.

##### Adverse events and safety evaluation

During the intervention period, the research team will continuously monitor and document all adverse events (AEs), including falls, dizziness, musculoskeletal pain, excessive fatigue, chest tightness, palpitations, and other untoward conditions occurring during or after exercise that require medical evaluation or management. Each AE will be recorded in terms of type, time of onset, duration, severity, management measures, and its relationship to the intervention. The severity of AEs will be classified into four grades: Grade 1 (mild)—symptoms are slight, do not affect daily living or training, and require no medical treatment; Grade 2 (moderate)—symptoms have a certain impact on daily activities or training and may require simple medical management; Grade 3 (severe)—symptoms result in significant functional impairment or inability to perform daily activities or training, necessitating evaluation or management by a medical institution; Grade 4 (serious)—including life-threatening conditions, fall-related fractures, hospitalization, or death. Serious adverse events (SAEs) will be reported promptly in accordance with the requirements of the research institution and the ethics committee. If acute dizziness, marked gait instability, sharp joint pain, chest tightness, palpitations, or other acute discomfort occurs during training, the exercise session will be immediately suspended, and a preliminary assessment will be conducted by the researchers. Intervention may resume only after symptoms have resolved and safety has been confirmed through evaluation. In the event of severe or serious AEs, recurrent falls, or newly developed contraindications to exercise, discontinuation of the intervention will be considered based on medical assessment. In cases of falls or other acute incidents, training will be halted immediately, and nursing home medical staff will be notified; emergency medical procedures will be initiated and family members contacted if necessary. Participants may withdraw their informed consent at any time. For participants who discontinue the intervention but consent to continue follow-up, every effort will be made to complete subsequent outcome assessments, and their data will be analyzed according to the pre-specified statistical analysis plan. The causal relationship between AEs and the intervention will be independently adjudicated by two researchers with clinical backgrounds who are not involved in the delivery of the intervention. The causality will be classified into five categories: definitely related, probably related, possibly related, unlikely related, and unrelated. In the event of disagreement, consensus will be reached through discussion. All AEs, along with their severity, causality, management measures, and any intervention pauses or terminations, will be documented. The incidence of AEs will be reported by group in the study results to evaluate the safety of the intervention.

##### Data collection and analysis

*Data collection*: After informed consent has been obtained from participants, baseline and follow-up assessments will be performed at four time points, T0, T1, T2 and T3, to collect study data. To ensure objective, accurate and consistent measurements, researchers will demonstrate standardised assessment procedures before the first measurement. All data collectors will receive unified standardised training and will be certified as proficient in the assessment methods. Following data collection, two evaluators will independently check the data and conduct double data entry to complete data input and archiving, thereby minimising entry bias.

*Data analysis*: Statistical analyses will be performed using SPSS version 27.0. Continuous variables will be presented as means ± standard deviations, while categorical variables will be presented as frequencies and percentages. Before statistical analysis, the distributional characteristics of the data will be assessed according to the nature of the variables. For baseline characteristics, continuous variables will be compared between groups using the independent-samples *t*-test or Mann–Whitney *U* test, as appropriate, and categorical variables will be compared using the chi-square test or Fisher’s exact test, as appropriate. For the primary outcome, the total SPPB score will be analysed using a linear mixed-effects model (LMM), with participant-specific random intercepts and group, time (T0, T1, T2, and T3), and group × time interaction as fixed effects. The primary endpoint will be week 12 (T3), and the primary effect estimate will be the between-group difference in the SPPB total score at week 12, with the between-group difference and its 95% confidence interval estimated using the LMM. The group × time interaction will be used to assess whether the pattern of change in SPPB over time differs between the intervention and control groups. Between-group comparisons at T1 and T2 will be conducted as supportive longitudinal analyses. Secondary outcomes will be prospectively classified as confirmatory secondary outcomes or exploratory outcomes according to the study objectives. Confirmatory secondary outcomes will include strength and balance ability, while cognitive function will be considered an exploratory outcome. Confirmatory secondary outcomes will be analysed using the same LMM approach as the primary outcome, with the primary comparison focusing on between-group differences at week 12 (T3). The Holm method will be used to adjust for multiple comparisons across the primary endpoint comparisons of the confirmatory secondary outcomes. No multiplicity adjustment will be applied to exploratory outcomes, and these results will be interpreted as hypothesis-generating. The classification of secondary outcomes and the corresponding multiplicity adjustment strategy will be prespecified before database lock. The primary analysis will follow the intention-to-treat (ITT) principle. For missing follow-up data, the LMM will be fitted under the assumption of missing at random (MAR), allowing participants with partially observed follow-up data to contribute to the analysis. The proportion of missing data, missing assessment time points, and reasons for missingness will be recorded and reported, and the pattern of missing data will be compared between the two groups. Prespecified sensitivity analyses will include per-protocol (PP) analysis and multiple imputation (MI) analysis to assess the robustness of the findings to protocol deviations and different approaches to handling missing data. If the pattern or reasons for missingness suggest that the MAR assumption may not hold, additional sensitivity analyses under prespecified missing-not-at-random (MNAR) scenarios will be conducted to assess the robustness of the findings under different assumptions regarding the missing-data mechanism. Exercise adherence will be analysed descriptively and expressed as percentages. Safety will be assessed by recording adverse events occurring during the intervention period. Adverse events will be reported separately for the intervention and control groups according to their frequency, type, severity, and potential relationship to the intervention. All statistical tests will be two-sided, with a significance level of *α* = 0.05. For the primary outcome and the confirmatory secondary outcomes after Holm adjustment, *p* < 0.05 will be considered statistically significant.

## Discussion

This study focuses on older adults with sarcopenia in nursing homes and designs an intervention programme of exercise snacks based on resistance training combined with Wuqinxi. It aims to explore a new sarcopenia intervention model that is scientific, safe and feasible. At present, the global ageing process is accelerating, and the proportion of older adults in China is continuously increasing. The high prevalence of sarcopenia in nursing homes is particularly prominent. Adverse outcomes caused by sarcopenia, such as falls and functional decline, not only seriously affect the quality of life of older adults but also increase the social and medical burden. Although the Asian Working Group for Sarcopenia Consensus has clearly established the core role of multimodal exercise, existing programmes still have deficiencies in adapting to the physical characteristics of older adults with sarcopenia and improving long-term adherence. This study is therefore based on this practical need.

The core strengths of this study lie in the innovative and integrated design of its intervention programme. First, in terms of exercise format, exercise snacks feature short bouts with frequent repetitions, which aligns well with the physiological characteristics of older adults with sarcopenia, such as weakened muscle strength and an inability to tolerate prolonged continuous exercise. This may address low adherence caused by excessive intensity and duration in traditional exercise programmes. Existing studies have confirmed that exercise snacks have high safety and feasibility among older adults and can effectively improve muscle function, balance ability and cognitive function. Furthermore, this study combines this emerging exercise mode with targeted training content for sarcopenia intervention, thereby expanding its application scope. Second, in terms of exercise content, the combined mode of resistance training plus Wuqinxi offers complementary advantages: as the core of sarcopenia intervention, resistance training directly increases muscle mass and strength, whereas Wuqinxi, with its gentle and continuous movement characteristics, improves balance ability and flexibility in older adults. This addresses the limitation of single-mode resistance training in insufficiently enhancing physical coordination, forming a dual intervention effect of muscle strengthening and functional improvement. Finally, this study introduces the Fogg Behaviour Model (FBM) to guide intervention implementation, constructing a support system across three dimensions: motivation activation, ability adaptation and prompt triggering. This directly addresses the difficulty of cultivating exercise habits in older adults with sarcopenia, provides theoretical support for the implementation and sustained advancement of the intervention programme, and enhances the scientific rigour and practical operability of the intervention.

This study also has certain limitations. First, it will be conducted in a single region, resulting in limited sample representativeness, which may restrict generalisability to all older adults with sarcopenia in nursing homes. Future studies could expand the sample size and include participants from different regions and types of nursing homes to verify the general applicability of the intervention programme. Second, a double-blind design is not feasible owing to the marked differences between the intervention and control conditions. Third, from the perspective of study design, this trial evaluates a comprehensive multi-component intervention package integrating exercise, health education, supervision, and personalised prompts. Accordingly, the between-group effects observed are attributable to the entire intervention package rather than the exercise component alone. Future studies may adopt an attention-matched control design, in which the control group receives researcher contact and general health-related activities with similar frequency and duration as the intervention group, to further control for the influences of attention effects and social interaction.

This study holds significant practical implications and theoretical value. In terms of practical significance, if the intervention is proven effective, it will provide older adults with sarcopenia a set of highly operable intervention tools. The exercise regimen primarily comprises resistance training and Wuqinxi, which entails low reliance on complex exercise equipment. Moreover, the intervention employs segmented, short-duration training sessions to reduce per-session exercise load, allows for individualized adjustments based on the physical condition of older adults, and thus demonstrates considerable potential for implementation. In terms of theoretical value, this study is the first to integrate resistance training, Wuqinxi, and exercise snacking, thereby enriching the content of multimodal exercise interventions for sarcopenia. Additionally, by applying the Fogg Behaviour Model (FBM) to promote exercise behaviour among older adults with sarcopenia, this study provides new empirical evidence for the application of behaviour change theories in exercise interventions for the older adults. Furthermore, the findings may serve as a reference for future research, for instance, in further optimizing the duration, frequency, and content composition of each exercise snacking session, or in exploring more efficient behavioural intervention strategies, thereby contributing to the continued refinement of a comprehensive intervention system for sarcopenia.

## Implications for the profession and/or patient care

If proven effective, this intervention may provide nurses working in long-term care settings with a feasible and low-cost strategy for sarcopenia management and healthy ageing promotion. The findings may support the development of evidence-based nurse-led exercise programmes for older adults residing in nursing homes.

## Impact

### What problem did this study address?

Sarcopenia is highly prevalent among nursing home residents and is associated with physical decline, falls and reduced quality of life. Existing exercise programmes often face challenges related to adherence and feasibility.

### What are the main findings?

This protocol is underpinned by the Fogg Behaviour Model and integrates resistance training, Wuqinxi and an exercise-snack approach. It aims to improve physical function among older adults with possible sarcopenia while enhancing exercise adherence and promoting the development of sustained exercise behaviours.

### Where and on whom will the research have an impact?

The findings may inform care for older adults with possible sarcopenia and may be relevant to nursing staff, rehabilitation professionals and long-term care institutions by providing evidence on a practical exercise-intervention model.
